# 1-{5-[(*E*)-(2-Fluoro­phen­yl)diazen­yl]-2-hy­droxy­phen­yl}ethanone

**DOI:** 10.1107/S1600536811004909

**Published:** 2011-02-16

**Authors:** Serap Yazıcı, Çiğdem Albayrak, Ismail Gümrükçüoğlu, Ismet Şenel, Orhan Büyükgüngör

**Affiliations:** a Department of Physics, Faculty of Arts and Sciences, Ondokuz Mayıs University, TR-55139 Kurupelit–Samsun, Turkey; b Sinop Faculty of Education, Sinop University, TR-57000 Sinop, Turkey; c Department of Chemistry, Ondokuz Mayıs University, TR-55139 Kurupelit–Samsun, Turkey

## Abstract

Theere are two independent mol­ecules in the asymmetric unit of the title compound, C_14_H_11_FN_2_O_2_, each with a *trans* configuration with respect to the azo double bond. The dihedral angle between the aromatic rings is 17.21 (2)° in one mol­ecule and 19.06 (2)° in the other. Each of the independent mol­ecules has an intra­molecular O—H⋯O hydrogen bond. In the crystal, mol­ecules are stacked along [100].

## Related literature

For general background to azo compounds, see: Catino & Farris (1985 ▶); Gregory (1991 ▶). For bond-length data, see: Allen *et al.* (1987 ▶); Deveci *et al.* (2005 ▶); Özdemir *et al.* (2006 ▶); Albayrak *et al.* (2009 ▶); Karabıyık *et al.* (2009 ▶); Yazıcı *et al.* (2011 ▶). 
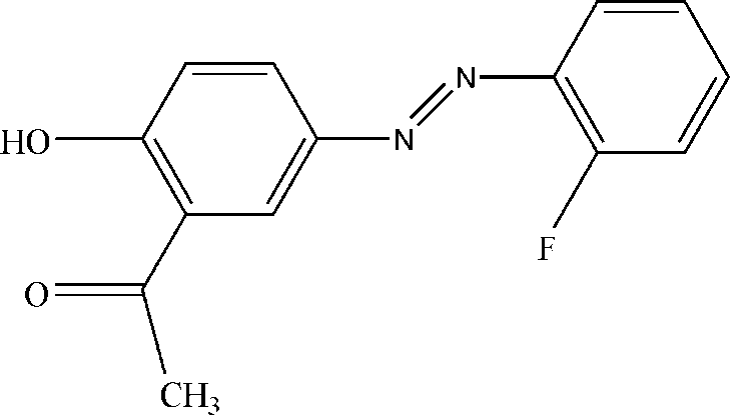


## Experimental

### 

#### Crystal data

C_14_H_11_FN_2_O_2_
*M* *_r_* = 258.25
Triclinic, 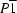

*a* = 6.7632 (3) Å
*b* = 12.5906 (6) Å
*c* = 13.8769 (6) Å
α = 85.641 (4)°
β = 89.337 (3)°
γ = 84.254 (4)°
*V* = 1172.31 (9) Å^3^
*Z* = 4
Mo *K*α radiation
μ = 0.11 mm^−1^
*T* = 150 K
0.64 × 0.40 × 0.12 mm

#### Data collection

Stoe IPDS II diffractometer
Absorption correction: integration (*X-RED32*; Stoe & Cie, 2002 ▶) *T*_min_ = 0.941, *T*_max_ = 0.987
20279 measured reflections
4870 independent reflections
3825 reflections with *I* > 2σ(*I*)
*R*_int_ = 0.034

#### Refinement

*R*[*F* ^2^ > 2σ(*F* ^2^)] = 0.036
*wR*(*F* ^2^) = 0.104
*S* = 1.04
4870 reflections
351 parameters
H atoms treated by a mixture of independent and constrained refinement
Δρ_max_ = 0.20 e Å^−3^
Δρ_min_ = −0.26 e Å^−3^

### 

Data collection: *X-AREA* (Stoe & Cie, 2002 ▶); cell refinement: *X-AREA*; data reduction: *X-RED32* (Stoe & Cie, 2002 ▶); program(s) used to solve structure: *SHELXS97* (Sheldrick, 2008 ▶); program(s) used to refine structure: *SHELXL97* (Sheldrick, 2008 ▶); molecular graphics: *ORTEP-3 for Windows* (Farrugia, 1997 ▶); software used to prepare material for publication: *WinGX* (Farrugia, 1999 ▶).

## Supplementary Material

Crystal structure: contains datablocks I, global. DOI: 10.1107/S1600536811004909/bh2334sup1.cif

Structure factors: contains datablocks I. DOI: 10.1107/S1600536811004909/bh2334Isup2.hkl

Additional supplementary materials:  crystallographic information; 3D view; checkCIF report

## Figures and Tables

**Table 1:** Hydrogen-bond geometry (Å, °)

*D*—H⋯*A*	*D*—H	H⋯*A*	*D*⋯*A*	*D*—H⋯*A*
O1*A*—H1*A*⋯O2*A*	0.91 (2)	1.68 (2)	2.5437 (12)	157 (2)
O1*B*—H1*B*⋯O2*B*	0.90 (2)	1.72 (2)	2.5395 (13)	150 (2)

**Fig. 1.**
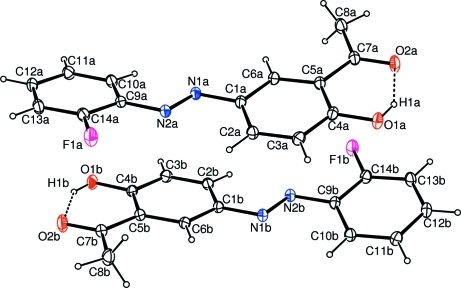
A view of the asymmetric unit of the title compound, with the atomic numbering scheme. Displacement ellipsoids are drawn at the 30% probability level.

## supplementary crystallographic information

### Comment

Azo dyes have been most widely used class of dyes due to its versatile applications in various fields, such as dyeing textile fibres, colouring different materials, plastics, biological-medical studies, electro-optical devices and ink-jet printers in high technology areas (Catino & Farris, 1985; Gregory, 1991).

The molecule of the title compound, with the atom numbering scheme, is shown in Fig. 1. The asymmetric unit contains two independent molecules (labelled *A* and *B*) with no significant differences in their structures. The conformations of the two molecules in the asymmetric unit are *trans* with respect to azo bridge. The dihedral angles between the aromatic rings are 17.21 (2)° for molecule *A* and 19.06 (2)° for molecule *B*. All bond lengths are in agreement with those reported for other azo compounds (Allen *et al.*, 1987; Deveci *et al.*, 2005; Özdemir *et al.*, 2006; Albayrak *et al.*, 2009; Karabıyık *et al.*, 2009; Yazıcı *et al.*, 2011). Each of the independent molecules has a strong intra-molecular O—H···O hydrogen bond which generates an *S*(6) ring motif. The crystal packing is stabilized by weak van der Waals interactions and molecules are stacked along crystallographic [100] direction.

### Experimental

A mixture of 2-fluoroaniline (0.86 g, 7.8 mmol), water (20 ml) and concentrated hydrochloric acid (1.97 ml, 23.4 mmol) was stirred until a clear solution was obtained. This solution was cooled down to 0–5 °C and a solution of sodium nitrite (0.75 g, 7.8 mmol) in water was added dropwise while the temperature was maintained below 5 °C. The resulting mixture was stirred for 30 min in an ice bath. 2-Hydroxyacetophenone (1.067 g, 7.8 mmol) solution (pH 9) was gradually added to a cooled solution of 2-fluorobenzenediazonium chloride, prepared as described above, and the resulting mixture was stirred at 0–5 °C for 2 h in an ice bath. The product was recrystallized from acetic acid to obtain solid (*E*)-2-acetyl-4-(2-fluorophenyldiazenyl)phenol. Crystals were obtained after one day by slow evaporation from benzene (yield 84%, m.p.= 414–416 K).

### Refinement

All C-bonded H atoms were placed in calculated positions and constrained to ride on their parents atoms, with C—H = 0.93–0.96 Å, and *U*_iso_(H) = 1.2*U*_eq_(C) or 1.5*U*_eq_(C). Hydroxyl H atoms were found in a difference map and refined freely (coordinates and isotropic displacement parameters).

### Crystal data

**Table:**

C_14_H_11_FN_2_O_2_	*Z* = 4
*M**_r_* = 258.25	*F*(000) = 536
Triclinic, *P*1	*D*_x_ = 1.463 Mg m^−^^3^
Hall symbol: -P 1	Melting point: 414 K
*a* = 6.7632 (3) Å	Mo *K*α radiation, λ = 0.71073 Å
*b* = 12.5906 (6) Å	Cell parameters from 26377 reflections
*c* = 13.8769 (6) Å	θ = 2.1–28.0°
α = 85.641 (4)°	µ = 0.11 mm^−^^1^
β = 89.337 (3)°	*T* = 150 K
γ = 84.254 (4)°	Prism, yellow
*V* = 1172.31 (9) Å^3^	0.64 × 0.40 × 0.12 mm

### Data collection

**Table:**

Stoe IPDS II diffractometer	4870 independent reflections
Radiation source: fine-focus sealed tube	3825 reflections with *I* > 2σ(*I*)
graphite	*R*_int_ = 0.034
rotation method scans	θ_max_ = 26.5°, θ_min_ = 2.1°
Absorption correction: integration (*X-RED32*; Stoe & Cie, 2002)	*h* = −8→8
*T*_min_ = 0.941, *T*_max_ = 0.987	*k* = −15→15
20279 measured reflections	*l* = −17→17

### Refinement

**Table:**

Refinement on *F*^2^	Primary atom site location: structure-invariant direct methods
Least-squares matrix: full	Secondary atom site location: difference Fourier map
*R*[*F*^2^ > 2σ(*F*^2^)] = 0.036	Hydrogen site location: inferred from neighbouring sites
*wR*(*F*^2^) = 0.104	H atoms treated by a mixture of independent and constrained refinement
*S* = 1.04	*w* = 1/[σ^2^(*F*_o_^2^) + (0.063*P*)^2^ + 0.1104*P*] where *P* = (*F*_o_^2^ + 2*F*_c_^2^)/3
4870 reflections	(Δ/σ)_max_ < 0.001
351 parameters	Δρ_max_ = 0.20 e Å^−^^3^
0 restraints	Δρ_min_ = −0.26 e Å^−^^3^
0 constraints	

### Fractional atomic coordinates and isotropic or equivalent isotropic displacement parameters (Å^2^)

**Table:**

	*x*	*y*	*z*	*U*_iso_*/*U*_eq_	
C7B	0.29323 (18)	0.16314 (9)	0.29768 (8)	0.0257 (3)	
C8B	0.3302 (2)	0.12570 (10)	0.19883 (9)	0.0330 (3)	
H8G	0.3581	0.0492	0.2033	0.049*	
H8H	0.4417	0.1579	0.1701	0.049*	
H8I	0.2146	0.1460	0.1596	0.049*	
F1B	0.19877 (12)	0.84085 (6)	0.20031 (5)	0.0356 (2)	
O1B	0.23372 (14)	0.24319 (8)	0.48420 (6)	0.0301 (2)	
O2B	0.30636 (15)	0.09721 (7)	0.36845 (6)	0.0377 (2)	
C1B	0.18273 (16)	0.46248 (9)	0.24684 (8)	0.0211 (2)	
C2B	0.15586 (17)	0.49461 (9)	0.34147 (8)	0.0228 (2)	
H2B	0.1256	0.5667	0.3513	0.027*	
C3B	0.17401 (17)	0.42033 (10)	0.41916 (8)	0.0248 (2)	
H3B	0.1573	0.4424	0.4815	0.030*	
C4B	0.21747 (17)	0.31175 (9)	0.40529 (8)	0.0230 (2)	
C5B	0.24380 (16)	0.27757 (9)	0.31097 (8)	0.0220 (2)	
C6B	0.22510 (16)	0.35533 (9)	0.23235 (8)	0.0213 (2)	
H6B	0.2415	0.3342	0.1697	0.026*	
C9B	0.15918 (16)	0.70366 (9)	0.09673 (8)	0.0207 (2)	
C10B	0.13857 (17)	0.67548 (9)	0.00216 (8)	0.0232 (2)	
H10B	0.1198	0.6053	−0.0089	0.028*	
C11B	0.14587 (18)	0.75119 (10)	−0.07505 (8)	0.0258 (3)	
H11B	0.1340	0.7313	−0.1378	0.031*	
C12B	0.17078 (18)	0.85670 (10)	−0.05954 (8)	0.0264 (3)	
H12B	0.1764	0.9070	−0.1119	0.032*	
C13B	0.18720 (18)	0.88725 (9)	0.03347 (9)	0.0269 (3)	
H13B	0.2022	0.9579	0.0445	0.032*	
C14B	0.18075 (17)	0.81039 (9)	0.10957 (8)	0.0241 (2)	
N1B	0.17444 (14)	0.53478 (8)	0.16294 (7)	0.0216 (2)	
N2B	0.16196 (14)	0.63179 (8)	0.18110 (7)	0.0223 (2)	
H1A	0.666 (3)	0.8198 (18)	0.0400 (15)	0.068 (6)*	
H1B	0.264 (3)	0.1779 (18)	0.4624 (15)	0.067 (6)*	
C1A	0.67173 (16)	0.53945 (9)	0.25268 (8)	0.0211 (2)	
C2A	0.65490 (17)	0.50686 (9)	0.15876 (8)	0.0231 (2)	
H2A	0.6472	0.4350	0.1496	0.028*	
C3A	0.64973 (17)	0.58043 (10)	0.08049 (8)	0.0241 (2)	
H3A	0.6396	0.5581	0.0185	0.029*	
C4A	0.65968 (16)	0.68882 (9)	0.09347 (8)	0.0224 (2)	
C5A	0.67659 (16)	0.72345 (9)	0.18736 (8)	0.0219 (2)	
C6A	0.68203 (16)	0.64637 (9)	0.26628 (8)	0.0212 (2)	
H6A	0.6927	0.6676	0.3286	0.025*	
C7A	0.69267 (17)	0.83745 (9)	0.19921 (8)	0.0249 (3)	
C8A	0.7238 (2)	0.87475 (10)	0.29742 (9)	0.0336 (3)	
H8D	0.7310	0.9507	0.2922	0.050*	
H8E	0.8455	0.8394	0.3241	0.050*	
H8F	0.6148	0.8580	0.3389	0.050*	
C9A	0.71928 (16)	0.29896 (9)	0.40370 (8)	0.0209 (2)	
C10A	0.69207 (17)	0.32845 (9)	0.49868 (8)	0.0239 (2)	
H10A	0.6558	0.3997	0.5099	0.029*	
C11A	0.71875 (18)	0.25244 (10)	0.57564 (8)	0.0268 (3)	
H11A	0.7033	0.2731	0.6384	0.032*	
C12A	0.76851 (19)	0.14535 (10)	0.56014 (9)	0.0282 (3)	
H12A	0.7863	0.0947	0.6125	0.034*	
C13A	0.79162 (19)	0.11398 (10)	0.46700 (9)	0.0289 (3)	
H13A	0.8229	0.0423	0.4559	0.035*	
C14A	0.76734 (18)	0.19117 (9)	0.39089 (8)	0.0256 (3)	
F1A	0.79319 (13)	0.16003 (6)	0.29986 (5)	0.0370 (2)	
N1A	0.68463 (14)	0.46776 (8)	0.33713 (7)	0.0217 (2)	
N2A	0.70257 (14)	0.37066 (8)	0.31933 (7)	0.0220 (2)	
O1A	0.65338 (13)	0.75669 (7)	0.01410 (6)	0.0286 (2)	
O2A	0.68122 (14)	0.90317 (7)	0.12833 (6)	0.0340 (2)	

### Atomic displacement parameters (Å^2^)

**Table:**

	*U*^11^	*U*^22^	*U*^33^	*U*^12^	*U*^13^	*U*^23^
C7B	0.0285 (6)	0.0206 (6)	0.0279 (6)	−0.0049 (4)	−0.0044 (5)	0.0035 (5)
C8B	0.0463 (8)	0.0207 (6)	0.0305 (6)	0.0038 (5)	−0.0045 (5)	−0.0012 (5)
F1B	0.0615 (5)	0.0232 (4)	0.0223 (4)	−0.0040 (3)	−0.0014 (3)	−0.0036 (3)
O1B	0.0421 (5)	0.0268 (5)	0.0208 (4)	−0.0070 (4)	−0.0022 (3)	0.0075 (3)
O2B	0.0598 (7)	0.0212 (5)	0.0309 (5)	−0.0048 (4)	−0.0045 (4)	0.0072 (4)
C1B	0.0206 (5)	0.0213 (6)	0.0211 (5)	−0.0039 (4)	−0.0013 (4)	0.0026 (4)
C2B	0.0258 (6)	0.0199 (5)	0.0228 (5)	−0.0034 (4)	0.0003 (4)	−0.0009 (4)
C3B	0.0261 (6)	0.0282 (6)	0.0204 (5)	−0.0047 (5)	0.0015 (4)	−0.0018 (4)
C4B	0.0224 (6)	0.0255 (6)	0.0209 (5)	−0.0069 (4)	−0.0022 (4)	0.0058 (4)
C5B	0.0227 (6)	0.0199 (6)	0.0233 (5)	−0.0046 (4)	−0.0019 (4)	0.0019 (4)
C6B	0.0235 (6)	0.0216 (6)	0.0190 (5)	−0.0045 (4)	−0.0011 (4)	0.0004 (4)
C9B	0.0195 (5)	0.0197 (6)	0.0216 (5)	0.0007 (4)	0.0020 (4)	0.0023 (4)
C10B	0.0262 (6)	0.0191 (5)	0.0239 (5)	−0.0005 (4)	0.0000 (4)	−0.0002 (4)
C11B	0.0291 (6)	0.0262 (6)	0.0211 (5)	0.0002 (5)	−0.0005 (4)	0.0003 (4)
C12B	0.0291 (6)	0.0223 (6)	0.0257 (6)	0.0006 (5)	0.0015 (4)	0.0068 (4)
C13B	0.0331 (7)	0.0169 (6)	0.0300 (6)	−0.0010 (4)	0.0004 (5)	0.0014 (5)
C14B	0.0288 (6)	0.0213 (6)	0.0216 (5)	0.0012 (4)	0.0001 (4)	−0.0020 (4)
N1B	0.0242 (5)	0.0182 (5)	0.0219 (5)	−0.0011 (4)	0.0000 (4)	0.0013 (4)
N2B	0.0236 (5)	0.0197 (5)	0.0229 (5)	−0.0012 (4)	0.0009 (4)	0.0016 (4)
C1A	0.0206 (5)	0.0207 (5)	0.0211 (5)	−0.0009 (4)	0.0009 (4)	0.0025 (4)
C2A	0.0251 (6)	0.0190 (5)	0.0248 (6)	−0.0012 (4)	0.0000 (4)	−0.0008 (4)
C3A	0.0255 (6)	0.0264 (6)	0.0198 (5)	0.0001 (4)	−0.0003 (4)	−0.0015 (4)
C4A	0.0192 (6)	0.0246 (6)	0.0216 (5)	0.0008 (4)	0.0013 (4)	0.0057 (4)
C5A	0.0213 (6)	0.0194 (6)	0.0240 (5)	−0.0003 (4)	0.0014 (4)	0.0018 (4)
C6A	0.0228 (6)	0.0208 (6)	0.0196 (5)	−0.0008 (4)	0.0012 (4)	−0.0004 (4)
C7A	0.0256 (6)	0.0208 (6)	0.0274 (6)	−0.0016 (4)	0.0020 (4)	0.0030 (5)
C8A	0.0492 (8)	0.0218 (6)	0.0306 (7)	−0.0081 (5)	−0.0015 (5)	−0.0002 (5)
C9A	0.0219 (6)	0.0198 (5)	0.0211 (5)	−0.0053 (4)	−0.0023 (4)	0.0023 (4)
C10A	0.0282 (6)	0.0191 (6)	0.0246 (6)	−0.0041 (4)	0.0006 (4)	−0.0003 (4)
C11A	0.0334 (7)	0.0263 (6)	0.0212 (5)	−0.0073 (5)	0.0000 (5)	−0.0001 (5)
C12A	0.0359 (7)	0.0233 (6)	0.0249 (6)	−0.0072 (5)	−0.0049 (5)	0.0073 (5)
C13A	0.0404 (7)	0.0171 (6)	0.0291 (6)	−0.0041 (5)	−0.0042 (5)	0.0012 (5)
C14A	0.0338 (7)	0.0220 (6)	0.0217 (6)	−0.0064 (5)	−0.0016 (4)	−0.0017 (4)
F1A	0.0663 (6)	0.0224 (4)	0.0221 (4)	−0.0024 (3)	−0.0016 (3)	−0.0040 (3)
N1A	0.0239 (5)	0.0192 (5)	0.0217 (5)	−0.0027 (4)	−0.0002 (4)	0.0015 (4)
N2A	0.0245 (5)	0.0191 (5)	0.0221 (5)	−0.0034 (4)	−0.0011 (4)	0.0016 (4)
O1A	0.0372 (5)	0.0261 (5)	0.0208 (4)	−0.0005 (4)	0.0009 (3)	0.0064 (3)
O2A	0.0500 (6)	0.0209 (4)	0.0298 (5)	−0.0046 (4)	−0.0003 (4)	0.0071 (4)

### Geometric parameters (Å, °)

**Table:**

C7B—O2B	1.2348 (14)	C1A—C6A	1.3822 (16)
C7B—C5B	1.4711 (16)	C1A—C2A	1.4051 (16)
C7B—C8B	1.4944 (17)	C1A—N1A	1.4217 (14)
C8B—H8G	0.9600	C2A—C3A	1.3712 (16)
C8B—H8H	0.9600	C2A—H2A	0.9300
C8B—H8I	0.9600	C3A—C4A	1.3984 (17)
F1B—C14B	1.3542 (13)	C3A—H3A	0.9300
O1B—C4B	1.3403 (13)	C4A—O1A	1.3400 (13)
O1B—H1B	0.90 (2)	C4A—C5A	1.4144 (16)
C1B—C6B	1.3802 (16)	C5A—C6A	1.4054 (15)
C1B—C2B	1.4076 (15)	C5A—C7A	1.4719 (16)
C1B—N1B	1.4203 (14)	C6A—H6A	0.9300
C2B—C3B	1.3707 (16)	C7A—O2A	1.2341 (14)
C2B—H2B	0.9300	C7A—C8A	1.4986 (17)
C3B—C4B	1.3966 (17)	C8A—H8D	0.9600
C3B—H3B	0.9300	C8A—H8E	0.9600
C4B—C5B	1.4125 (16)	C8A—H8F	0.9600
C5B—C6B	1.4074 (15)	C9A—C14A	1.3879 (16)
C6B—H6B	0.9300	C9A—C10A	1.4015 (16)
C9B—C14B	1.3911 (16)	C9A—N2A	1.4214 (14)
C9B—C10B	1.3974 (15)	C10A—C11A	1.3796 (16)
C9B—N2B	1.4234 (14)	C10A—H10A	0.9300
C10B—C11B	1.3827 (16)	C11A—C12A	1.3889 (17)
C10B—H10B	0.9300	C11A—H11A	0.9300
C11B—C12B	1.3887 (17)	C12A—C13A	1.3825 (17)
C11B—H11B	0.9300	C12A—H12A	0.9300
C12B—C13B	1.3833 (17)	C13A—C14A	1.3795 (16)
C12B—H12B	0.9300	C13A—H13A	0.9300
C13B—C14B	1.3804 (16)	C14A—F1A	1.3551 (13)
C13B—H13B	0.9300	N1A—N2A	1.2593 (14)
N1B—N2B	1.2603 (14)	O1A—H1A	0.91 (2)
			
O2B—C7B—C5B	120.13 (11)	C6A—C1A—C2A	119.80 (10)
O2B—C7B—C8B	119.40 (11)	C6A—C1A—N1A	116.42 (10)
C5B—C7B—C8B	120.47 (10)	C2A—C1A—N1A	123.76 (10)
C7B—C8B—H8G	109.5	C3A—C2A—C1A	120.38 (11)
C7B—C8B—H8H	109.5	C3A—C2A—H2A	119.8
H8G—C8B—H8H	109.5	C1A—C2A—H2A	119.8
C7B—C8B—H8I	109.5	C2A—C3A—C4A	120.28 (10)
H8G—C8B—H8I	109.5	C2A—C3A—H3A	119.9
H8H—C8B—H8I	109.5	C4A—C3A—H3A	119.9
C4B—O1B—H1B	105.8 (13)	O1A—C4A—C3A	117.37 (10)
C6B—C1B—C2B	119.67 (10)	O1A—C4A—C5A	122.32 (11)
C6B—C1B—N1B	116.49 (10)	C3A—C4A—C5A	120.32 (10)
C2B—C1B—N1B	123.81 (10)	C6A—C5A—C4A	118.24 (10)
C3B—C2B—C1B	120.40 (11)	C6A—C5A—C7A	122.30 (10)
C3B—C2B—H2B	119.8	C4A—C5A—C7A	119.44 (10)
C1B—C2B—H2B	119.8	C1A—C6A—C5A	120.98 (10)
C2B—C3B—C4B	120.37 (11)	C1A—C6A—H6A	119.5
C2B—C3B—H3B	119.8	C5A—C6A—H6A	119.5
C4B—C3B—H3B	119.8	O2A—C7A—C5A	120.32 (11)
O1B—C4B—C3B	117.47 (10)	O2A—C7A—C8A	119.38 (11)
O1B—C4B—C5B	122.31 (11)	C5A—C7A—C8A	120.30 (10)
C3B—C4B—C5B	120.22 (10)	C7A—C8A—H8D	109.5
C6B—C5B—C4B	118.39 (10)	C7A—C8A—H8E	109.5
C6B—C5B—C7B	122.06 (10)	H8D—C8A—H8E	109.5
C4B—C5B—C7B	119.54 (10)	C7A—C8A—H8F	109.5
C1B—C6B—C5B	120.95 (10)	H8D—C8A—H8F	109.5
C1B—C6B—H6B	119.5	H8E—C8A—H8F	109.5
C5B—C6B—H6B	119.5	C14A—C9A—C10A	117.45 (10)
C14B—C9B—C10B	117.45 (10)	C14A—C9A—N2A	117.30 (10)
C14B—C9B—N2B	117.23 (10)	C10A—C9A—N2A	125.26 (10)
C10B—C9B—N2B	125.31 (10)	C11A—C10A—C9A	120.39 (11)
C11B—C10B—C9B	120.50 (11)	C11A—C10A—H10A	119.8
C11B—C10B—H10B	119.7	C9A—C10A—H10A	119.8
C9B—C10B—H10B	119.7	C10A—C11A—C12A	120.57 (11)
C10B—C11B—C12B	120.42 (11)	C10A—C11A—H11A	119.7
C10B—C11B—H11B	119.8	C12A—C11A—H11A	119.7
C12B—C11B—H11B	119.8	C13A—C12A—C11A	120.07 (11)
C13B—C12B—C11B	120.26 (11)	C13A—C12A—H12A	120.0
C13B—C12B—H12B	119.9	C11A—C12A—H12A	120.0
C11B—C12B—H12B	119.9	C14A—C13A—C12A	118.64 (11)
C14B—C13B—C12B	118.46 (11)	C14A—C13A—H13A	120.7
C14B—C13B—H13B	120.8	C12A—C13A—H13A	120.7
C12B—C13B—H13B	120.8	F1A—C14A—C13A	118.30 (11)
F1B—C14B—C13B	118.04 (11)	F1A—C14A—C9A	118.85 (10)
F1B—C14B—C9B	119.08 (10)	C13A—C14A—C9A	122.85 (11)
C13B—C14B—C9B	122.87 (11)	N2A—N1A—C1A	113.45 (9)
N2B—N1B—C1B	113.68 (9)	N1A—N2A—C9A	113.47 (9)
N1B—N2B—C9B	113.37 (9)	C4A—O1A—H1A	101.3 (13)
			
C6B—C1B—C2B—C3B	0.82 (17)	C6A—C1A—C2A—C3A	0.36 (17)
N1B—C1B—C2B—C3B	−177.35 (10)	N1A—C1A—C2A—C3A	−177.85 (10)
C1B—C2B—C3B—C4B	−0.62 (17)	C1A—C2A—C3A—C4A	−0.48 (17)
C2B—C3B—C4B—O1B	179.85 (10)	C2A—C3A—C4A—O1A	−179.82 (10)
C2B—C3B—C4B—C5B	0.22 (17)	C2A—C3A—C4A—C5A	0.46 (17)
O1B—C4B—C5B—C6B	−179.62 (10)	O1A—C4A—C5A—C6A	179.99 (10)
C3B—C4B—C5B—C6B	−0.01 (17)	C3A—C4A—C5A—C6A	−0.30 (16)
O1B—C4B—C5B—C7B	−0.76 (17)	O1A—C4A—C5A—C7A	−1.47 (16)
C3B—C4B—C5B—C7B	178.86 (10)	C3A—C4A—C5A—C7A	178.24 (10)
O2B—C7B—C5B—C6B	−179.27 (11)	C2A—C1A—C6A—C5A	−0.21 (17)
C8B—C7B—C5B—C6B	1.43 (17)	N1A—C1A—C6A—C5A	178.13 (10)
O2B—C7B—C5B—C4B	1.91 (17)	C4A—C5A—C6A—C1A	0.18 (16)
C8B—C7B—C5B—C4B	−177.39 (11)	C7A—C5A—C6A—C1A	−178.31 (10)
C2B—C1B—C6B—C5B	−0.62 (17)	C6A—C5A—C7A—O2A	−177.81 (11)
N1B—C1B—C6B—C5B	177.68 (10)	C4A—C5A—C7A—O2A	3.72 (17)
C4B—C5B—C6B—C1B	0.21 (17)	C6A—C5A—C7A—C8A	2.39 (17)
C7B—C5B—C6B—C1B	−178.62 (10)	C4A—C5A—C7A—C8A	−176.08 (11)
C14B—C9B—C10B—C11B	1.95 (16)	C14A—C9A—C10A—C11A	1.94 (17)
N2B—C9B—C10B—C11B	−177.37 (10)	N2A—C9A—C10A—C11A	−177.74 (10)
C9B—C10B—C11B—C12B	−0.98 (17)	C9A—C10A—C11A—C12A	−1.46 (18)
C10B—C11B—C12B—C13B	−0.44 (18)	C10A—C11A—C12A—C13A	−0.06 (19)
C11B—C12B—C13B—C14B	0.81 (18)	C11A—C12A—C13A—C14A	1.01 (19)
C12B—C13B—C14B—F1B	179.40 (10)	C12A—C13A—C14A—F1A	179.01 (11)
C12B—C13B—C14B—C9B	0.23 (18)	C12A—C13A—C14A—C9A	−0.48 (19)
C10B—C9B—C14B—F1B	179.24 (10)	C10A—C9A—C14A—F1A	179.53 (10)
N2B—C9B—C14B—F1B	−1.38 (16)	N2A—C9A—C14A—F1A	−0.77 (16)
C10B—C9B—C14B—C13B	−1.59 (17)	C10A—C9A—C14A—C13A	−0.98 (18)
N2B—C9B—C14B—C13B	177.78 (11)	N2A—C9A—C14A—C13A	178.72 (11)
C6B—C1B—N1B—N2B	−170.98 (10)	C6A—C1A—N1A—N2A	−170.56 (10)
C2B—C1B—N1B—N2B	7.24 (16)	C2A—C1A—N1A—N2A	7.71 (16)
C1B—N1B—N2B—C9B	178.39 (9)	C1A—N1A—N2A—C9A	178.87 (9)
C14B—C9B—N2B—N1B	−169.03 (10)	C14A—C9A—N2A—N1A	−171.20 (10)
C10B—C9B—N2B—N1B	10.29 (16)	C10A—C9A—N2A—N1A	8.47 (16)

### Hydrogen-bond geometry (Å, °)

**Table:**

*D*—H···*A*	*D*—H	H···*A*	*D*···*A*	*D*—H···*A*
O1A—H1A···O2A	0.91 (2)	1.68 (2)	2.5437 (12)	157 (2)
O1B—H1B···O2B	0.90 (2)	1.72 (2)	2.5395 (13)	150 (2)
